# Field demonstration of a semiochemical treatment that enhances *Diorhabda carinulata* biological control of *Tamarix* spp.

**DOI:** 10.1038/s41598-019-49459-5

**Published:** 2019-09-10

**Authors:** Alexander M. Gaffke, Sharlene E. Sing, Tom L. Dudley, Daniel W. Bean, Justin A. Russak, Agenor Mafra-Neto, Robert K. D. Peterson, David K. Weaver

**Affiliations:** 1 Department of Land Resources and Environmental Sciences, Montana State University, Bozeman, MT 59717 USA; 2 Agricultural Research Service, United States Department of Agriculture, Center for Medical, Agricultural, and Veterinary Entomology, Gainesville, FL 32608 USA; 3 USDA Forest Service, Rocky Mountain Research Station, Bozeman, MT 59717 USA; 4 Marine Science Institute, University of California, Santa Barbara, CA 93106 USA; 5 Colorado Department of Agriculture, Palisade Insectary, Palisade, CO 81526 USA; 6 Department of Chemistry and Biochemistry, University of California Santa Barbara, Santa Barbara, CA 93106 USA; 7 ISCA Technologies, Inc., Riverside, CA 92507 USA

**Keywords:** Entomology, Invasive species

## Abstract

The northern tamarisk beetle *Diorhabda carinulata* (Desbrochers) was approved for release in the United States for classical biological control of a complex of invasive saltcedar species and their hybrids (*Tamarix* spp.). An aggregation pheromone used by *D. carinulata* to locate conspecifics is fundamental to colonization and reproductive success. A specialized matrix formulated for controlled release of this aggregation pheromone was developed as a lure to manipulate adult densities in the field. One application of the lure at onset of adult emergence for each generation provided long term attraction and retention of *D. carinulata* adults on treated *Tamarix* spp. plants. Treated plants exhibited greater levels of defoliation, dieback and canopy reduction. Application of a single, well-timed aggregation pheromone treatment per generation increased the efficacy of this classical weed biological control agent.

## Introduction

The genus *Tamarix* (Tamaricaceae) are invasive Eurasian woody trees or shrubs increasingly present and dominant in riparian areas of the western United States^1–4^. Multiple *Tamarix* species, collectively referred to as saltcedar or tamarisk, are present in the United States, with widespread hybridization between the species^3^. To simplify the discussion of this species complex, *Tamarix* species and their hybrids will hereafter be referred to as *Tamarix*. Since its introduction, *Tamarix* has significantly degraded native plant communities and wildlife habitat through the replacement of native plant assemblages with monocultures^4^. The loss of native plant assemblages from *Tamarix* invasion imperils multiple populations of threatened or endangered species^2^. The loss of ecosystem services and functions due to *Tamarix* invasion has been estimated between $133–185 million U.S. dollars annually^5,6^. The disruption of ecological processes caused by *Tamarix* ecosystem dominance was the impetus for the development of a classical biological control program for *Tamarix*, utilizing the northern tamarisk beetle *Diorhabda carinulata* Desbrochers (formerly *D. elongata deserticola* Brullé) (Coleoptera: Chrysomelidae)^7,8^. After extensive host-specificity testing, *D. carinulata* was found to be host specific to *Tamarix* and did not pose a risk to native plants, leading to the approval and introduction of *D. carinulata* to the United States^7,8^.

In North America, *D. carinulata* populations are multivoltine, with 1–3 generations per year^9,10^. *Diorhabda carinulata* overwinters in the adult stage and emerges in spring, coinciding with *Tamarix* bud break^10^. After spring emergence, adults feed and mate followed by the deposition of clutches of eggs onto *Tamarix* foliage^9^. Once the eggs hatch, the three larval stages feed within the *Tamarix* canopy then descend into the understory litter to form pupal cells^10^. After metamorphosis, the adults emerge from the litter and disperse both short and long distances^11^. Along with the dispersal behavior, the adults aggregate on *Tamarix* plants^12^. In response to shorter day length at the approach of autumn, *D. carinulata* adults enter a reproductive diapause but continue feeding to accumulate metabolic reserves, after which they burrow into the litter to overwinter^13^.

All life stages of *D. carinulata* feed exclusively on *Tamarix* foliage. Feeding by *D. carinulata* abrades the *Tamarix* foliar cuticle; widespread cuticular disruption by high densities of beetles results in desiccation of the foliage, leading to partial or complete host defoliation^14^. Defoliation by *D. carinulata* can dramatically increase the level of physiological stress experienced by affected plants and depletes reserves of stored carbon^15–17^. This diminishes foliage production and root mass in the years following a defoliation event^16^. Multiple years of intense herbivory by *D. carinulata* can result in *Tamarix* mortality^16,18^.

Techniques to manipulate densities of *D. carinulata* in the field have recently been investigated, specifically to increase the biocontrol efficacy of sparse beetle populations^19^. These manipulations involved the use of *D. carinulata*’s aggregation pheromone (2*E*,4*Z*)-2,4-heptadien-1-ol, which was identified by Cossé *et al*.^12,19,20^. Treatments incorporating semiochemicals applied through a specialized pheromone lure application technology (SPLAT^®^: ISCA Technologies, Riverside, CA, USA) made it possible to create or enhance aggregations of adults and larvae. Weekly application of 1-g doses of SPLAT impregnated with aggregation-causing semiochemicals (2.17% (2*E*,4*Z*)-2,4-heptadien-1-ol) resulted in persistent and higher densities of *D. carinulata* on treated plants. Higher densities resulted in intensified herbivory with increased rates of defoliation, plant dieback, and reduction in live canopy volume. These are desirable outcomes for a classical weed biological control program. Along with intensifying impact on *Tamarix*, the ability to aggregate populations of a biological control agent in the field is also useful for facilitating monitoring (e.g., to track presence/absence, persistence, life stage or abundance) by biological control practitioners^12,20,21^.

Recently published results show that the release of the aggregation pheromone attained from 1-g doses of formulated SPLAT maintained aggregations of *D. carinulata* for only 7–10 days^19^. The limited duration of lure activity was due to rapid volatilization of the aggregation pheromone from the small volume of the dollops used in the 1-g application. The restricted bioactivity of 1-g treatments significantly constrains the practical utility of this technology. Treatments based on a 1-g lure required weekly applications throughout the summer to maintain consistent aggregations of *D. carinulata*^18^. The activity of SPLAT-based pheromone treatments can be extended by adjusting the volume applied^22^. An increased application rate of pheromone impregnated SPLAT was therefore investigated using larger dollops. The objective of this study was to determine if three applications of 4-g doses of SPLAT targeting discrete adult emergence in this multivoltine beetle could result in season-long manipulation of *D. carinulata* densities. The rationale for evaluating targeted application of larger volumes of SPLAT-based semiochemical lures was to determine if longer term aggregations could be maintained, therefore reducing the time commitment required for practical deployment of this technique by land managers.

## Results

### Release rate analysis

Release rates of *D. carinulata* aggregation pheromone (2*E*,4*Z*)-2,4-heptadien-1-ol from 4-g dollops of field-aged SPLAT over a 31-day period are displayed in Fig. S1. The release rate model y = 19384x^−2.3^ yielded an R^2^ of 0.93 and a decay constant of −2.3. On day 1, mean *D. carinulata* aggregation pheromone emitted from treated dollops was 17514 nghr^−1^. Pheromone emissions from the 4-g dollops rapidly decreased between the first and second day of volatile collection, but stabilized after day 15. Between day 15 and 31, the pheromone was released at a mean rate of 228 nghr^−1^.

### Field results

#### Reproductive adults

**Figure 1:**
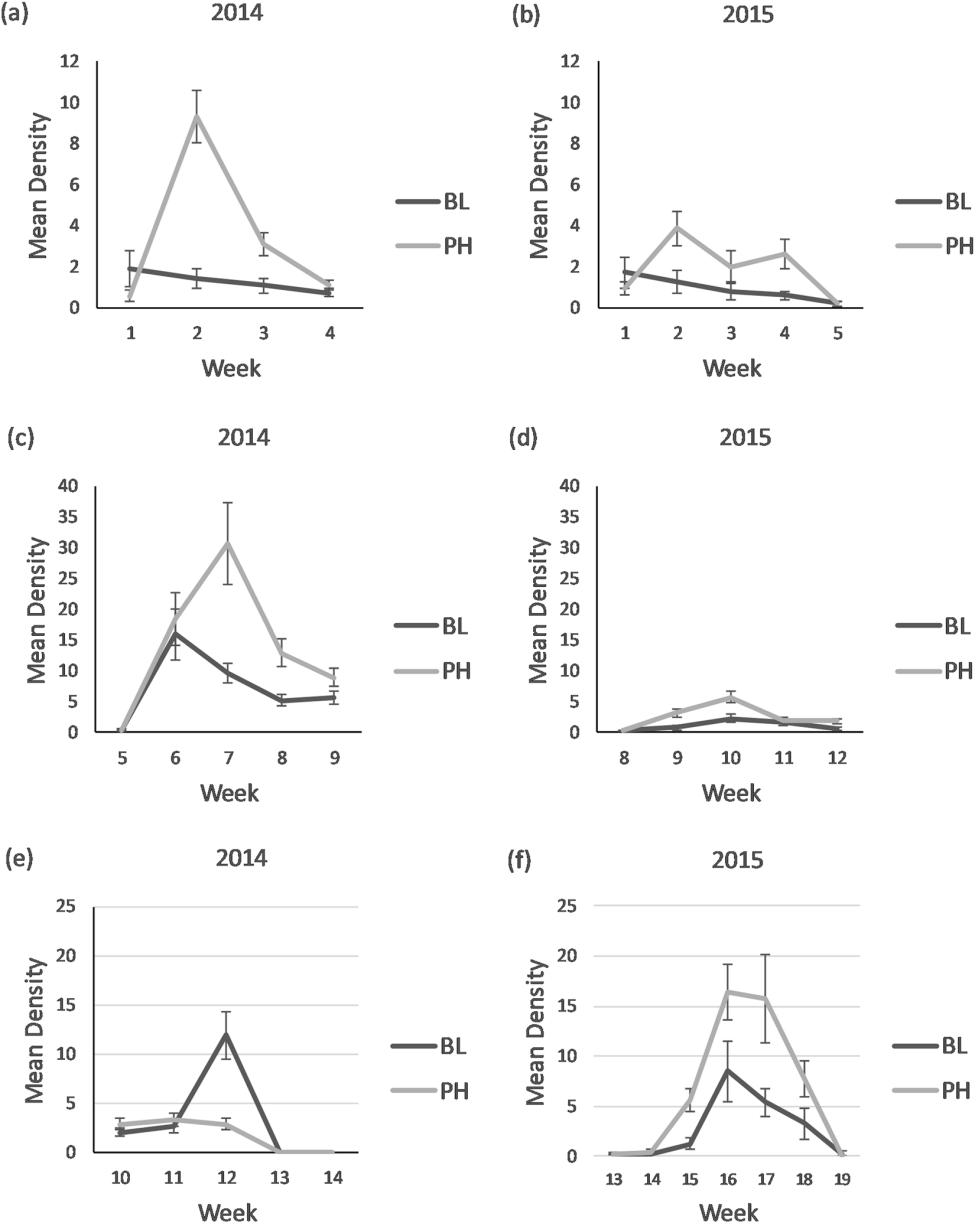
Weekly mean ± SE capture per three sweeps of adult *Diorhabda carinulata* on plants receiving 4-g dollops of pheromone impregnated SPLAT (PH) or blank SPLAT (BL). Densities captured 2014–15 of P generation overwintered adults (**a**,**b**), F1 reproductive adults (**c,d**), and F2 non-reproductive adults (**e,f**). Overall the pheromone effect was significant and greater for (**a**) (F_1,185.2_ = 21.32, *P* < 0.001), (**b**) (F_1,186.3_ = 12.3, *P* < 0.001), (**c**) (F_1,232.1_ = 20, *P* < 0.001), (**d**) (F_1,208.3_ = 20.1, *P* < 0.001), and (**f**) (F_1,348_ = 31, *P* < 0.001). There was no overall pheromone effect for (**e**) (F_1,234.4_ = 1.2, *P* = 0.28).

In 2014, weekly sweeps of recently emerged over-wintering (P) adult *D. carinulata* on *Tamarix* fitted with treatment lures yielded a mean of 1.3 on plants receiving the BL (blank or control) treatment, compared to 3.5 on plants receiving the PH (aggregation pheromone) treatment (F_1,185.2_ = 21.32, *P* < 0.001) (Fig. 1a). The 2014 weekly sweeps of F1 adults resulted in overall greater mean densities than the sweeps of 2014 P generation adults, again with higher densities swept from plants treated with the pheromone lure, 7.3 from BL-treated plants vs. 14.3 from PH-treated plants (F_1,232.1_ = 20, *P* < 0.001) (Fig. 1c). There was a significant site effect for F1 adult numbers in 2014 (F_1,232.1_ = 10.7, *P* = 0.001 on 1, 232.1 d.f.); however, a site x treatment interaction was not found to be significant (F_1,232.1_ = 20, *P* = 0.58).

Mean weekly sweep captures of overwintered P adults in 2015 were lower than recorded in 2014, averaging 1.1 adults on BL-treated plants vs. 2.2 adults on PH-treated plants (F_1,186.3_ = 12.3, *P* < 0.001) (Fig. 1b). Counts of the 2015 F1 adults were also lower than reported for 2014 F1 adults, averaging 1.2 and 2.7 adults per three sweeps, respectively, on BL-treated vs. PH-treated plants (F_1,208.3_ = 20.1, *P* < 0.001) (Fig. 1d). There was no significant site effect for overwintered P or F1 adult sweep capture in 2015 (F_1,188_ = 1.6, *P* = 0.21; F _1,211.4_ = 2.9, *P* = 0.09).

#### Non-reproductive adults

No pheromone-mediated difference in weekly sweep captures of F2 adults occurred in 2014 (F_1,234.4_ = 1.2, *P* = 0.28) (Fig. 1e). There was a significant site effect (F_1,234.4_ = 7, *P* = 0.008), but the interaction between site and treatment for F2 adults was not significant (F_1,234.4_ = 1, *P* = 0.32). In 2015, sweeps of PH-treated plants yielded significantly higher densities of F2 adults, averaging 6.6 adults compared to a mean of 2.7 adults on BL-treated plants (F_1,348_ = 31, *P* < 0.001) (Fig. 1f). There was a significant site effect in 2015 (F_1,350.3_ = 5.5, *P* = 0.02), and an ordinal interaction between site and treatment (F_1,348_ = 6.2, *P* = 0.01).

#### Larval densities

**Figure 2:**
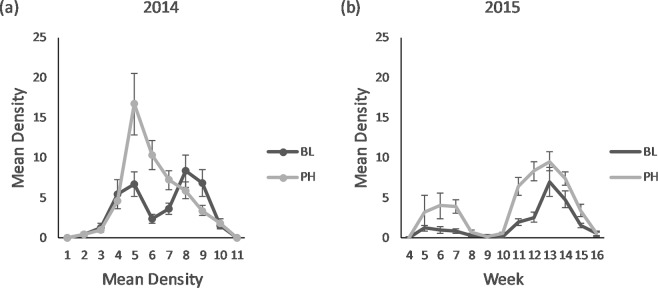
Weekly mean ± SE larval *Diorhabda carinulata* capture per three sweeps on plants receiving 4-g dollops of pheromone impregnated SPLAT (PH) or 4 g dollops of blank SPLAT (BL), in 2014 (**a**), and 2015 (**b**). Overall pheromone effect was significant and greater for (**a**) (F_1,467_ = 7.2, *P* = 0.007), and (**b**) (F_1,514.1_ = 45.3, *P* < 0.001).

Increased densities of adult *D. carinulata* recorded on PH-treated plants were correlated with increased mean densities of larvae captured in weekly sweeps of the same plants (Fig. 2). In 2014, the mean number of larvae captured per three sweeps each week averaged 5.1 on PH-treated plants, compared to 3.7 on BL-treated plants (F_1,467_ = 7.2, *P* = 0.007) (Fig. 2a). There was no site effect for numbers of larvae in 2014 (F_1,467_ = 1.4, *P* = 0.23); however, there was a significant ordinal interaction between treatment and site (F_1,467_ = 3.9, *P* = 0.05). Densities of larvae captured in weekly sweeps during 2015 were lower overall, but the numbers were still greater on PH-treated compared to BL-treated plants, averaging 3.2 vs. 1.5, respectively (F_1,514.1_ = 45.3, *P* < 0.001) (Fig. 2b).

#### Damage, dieback and canopy volume

**Figure 3:**
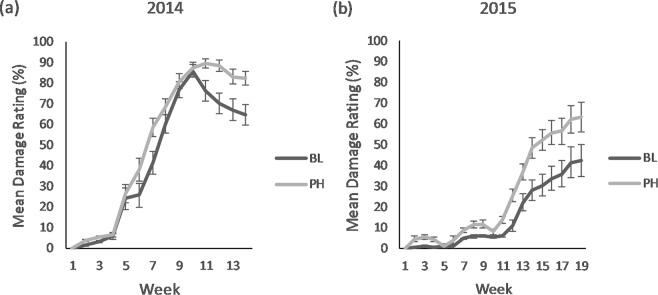
Weekly mean ± SE damage rating for *Tamarix* plants receiving 4-g dollops of pheromone impregnated SPLAT (PH) or blank SPLAT (BL), in 2014 (**a**) and 2015 (**b**). Overall pheromone effect was significant and greater for (**a**) (F_1,655_ = 34.8, *P* < 0.001), and (**b**) (F_1,799.1_ = 76.5, *P* < 0.001).

In 2014, ratings of *D. carinulata* feeding damage for PH-treated plants differed significantly from damage ratings reported for BL-treated plants (F_1,655_ = 34.8, *P* < 0.001) (Fig. 3a). Although all monitored plants (i.e., PH and BL) experienced nearly complete defoliation during summer 2014, in-season regrowth after defoliation reported for BL-treated plants was significantly greater compared with regrowth after defoliation reported for PH-treated plants (Fig. 3a). Again in 2015, PH-treated plants had greater feeding damage than BL-treated plants (F_1,799.1_ = 76.5, *P* < 0.001) (Fig. 3b). No significant in-season regrowth was observed in 2015.

**Figure 4:**
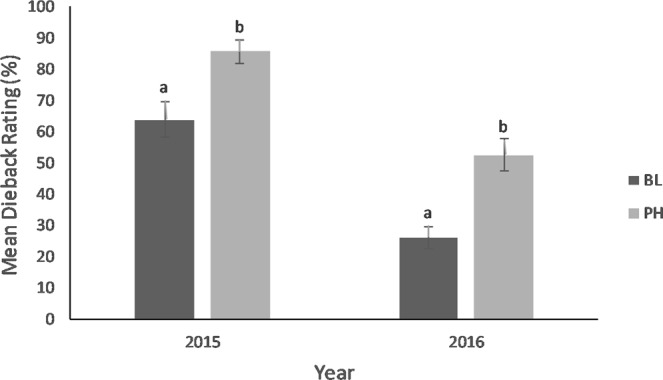
Mean ± SE dieback rating for *Tamarix* plants receiving 4-g dollops of pheromone impregnated SPLAT (PH) or blank SPLAT (BL) in 2015 and 2016. Different letters above error bars denote significant differences between treatments.

**Figure 5:**
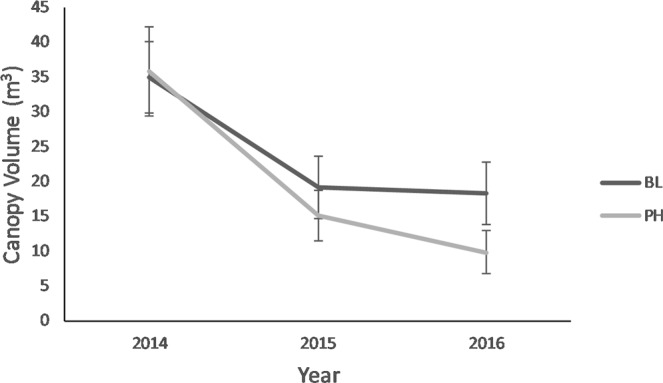
Change in canopy volume 2014–2016 for *Tamarix* plants receiving 4-g dollops of pheromone impregnated SPLAT (PH) or blank SPLAT (BL). Overall pheromone effect was significant and greater (F_1,140_ = 6.3, *P* = 0.01).

Increased feeding damage recorded on PH-treated plants was reflected in increased dieback observed in the following year, both in 2015 (F_1,44_ = 10, *P* = 0.003) and 2016 (F_1,43_ = 17.5, *P* < 0.001) (Fig. 4). Dieback was greater for both the PH-treated and BL-treated plants in 2015 compared to 2016, most likely due to the greater level of defoliation recorded across the study site in 2015 compared to 2016. Canopy volume did not significantly differ one year after defoliation. In the second year after defoliation, PH-treated plants had smaller canopy volumes compared with study plants receiving a second season of BL treatment (F_1,140_ = 6.3, *P* = 0.01) (Fig. 5).

## Discussion

The use of synthetic aggregation pheromones to manipulate the spatial distribution of biological control agents has been suggested as a method to intensify damage on under-exploited weed infestations^23^.The results of this investigation confirm that the deployment of an aggregation pheromone is a feasible approach for manipulating the density and spatial distribution of *D. carinulata* to enhance biological control of *Tamarix*. Effective spatial manipulations and enhanced herbivory were achieved with only three targeted applications of the aggregation pheromone per year. The ability of biological control practitioners to alter densities of a classical biological control agent weeks after an application of an attractive lure could have broad impacts in the field of biological control.

Emissions of synthetic (2*E*,4*Z*)-2,4-heptadien-1-ol, the *D. carinulata* aggregation pheromone, from field deployed 4-g dollops of SPLAT were variable for the first 5 days after exposure to environmental conditions. Emission rates stabilized at 10 days and remained relatively constant over the following 21 days, until the study was terminated on day 31. On the first day, the hourly amount of synthetic aggregation pheromone emitted from 4-g dollops approached the amount estimated to be produced by 3,300 adult *D. carinulata* males in one hour, based on the reported emission rate of 5.2 ng/male/hour^12^. The average pheromone release rate from the 4-g dollops between 10 and 31 days was equivalent to estimated hourly pheromone emissions by 60 adult male *D. carinulata*^12^.

Differences in captures of reproductive adult beetles on *Tamarix* receiving the control vs. pheromone treatment indicates that a single application of a 4-g dollop of SPLAT impregnated with (2*E*,4*Z*)-2,4-heptadien-1-ol can maintain local aggregations of adult *D. carinulata* over the course of their generational life span (Fig. 1)^10^. The response of the non-reproductive F2 adults was not consistent across the two years of this study. No pheromone mediated changes in density were observed in 2014 but increased F2 adult densities were recorded in 2015 (Fig. 1e,f).

Increased rates of damage, dieback, and greater changes in canopy volume were correlated with higher *D. carinulata* densities. Damage ratings for monitored trees varied greatly across the years. In 2014, all of the monitored plants, regardless of the treatment received, were nearly completely defoliated by week 10, with observable damage occurring primarily after week 5. After week 10, the BL-treated plants had substantially higher rates of regrowth compared to the PH-treated plants, indicated by a decrease in mean damage rating for BL-treated plants (Fig. 3a). This suggests that the increased damage rating for PH-treated plants earlier in the season is indicative of the influence of the pheromone treatment on the timing or intensity of herbivory, which impairs the ability of PH-treated plants to regrow after defoliation.

Higher in-season feeding damage ratings reported for all study plants in 2014 were correlated with the greater rate of dieback on all study plants recorded in 2015. Differences in canopy volume were, in contrast to the more immediate influence of damage on dieback, only detected after plants experienced a second year of defoliation. However, the limited but targeted application of the *D. carinulata* aggregation pheromone resulted in a mean 73% reduction in canopy volume in only two years. This result was unexpected because *Tamarix* exposed to two years of defoliation by *Diorhabda* spp. typically experiences a 25–33% reduction in canopy volume^24^.

Application of *D. carinulata* aggregation pheromone based lures effectively allows for land managers to dictate where *D. carinulata* feeding damage will occur. This would make pheromone formulations a very useful tool in managing *Tamarix* stands that have not been sufficiently defoliated to cause canopy die back or mortality. Reports in which impact due to herbivory has been measured show a high degree of variation in die-back and mortality from site to site^25^. In one study mortality was measured at nearly 80% at a site that had been defoliated multiple times, while at a neighboring site two kilometers away, which had been more sporadically defoliated, *Tamarix* mortality was only 20%^11^. In such instances pheromone formulations could be used to attract and concentrate beetles at areas with low mortality or die back. There are areas in western Colorado where *D. carinulata* has been present for ten years, and where impact has been minimal^25^. Landowners would probably welcome the use of pheromone-based lures as an additional management tool to increase the impact of *Diorhabda* spp. on *Tamarix*. Further research and development will be needed to finalize the production of a commercially viable pheromone lure system for *D. carinulata*. We expect that this efficient low-environmental impact herbivore aggregation solution for *Tamarix* management will become commercially available and widely adopted, but the total cost for the treatment of an individual tree will depend on the level of adoption and commercialization of the technology. A similar system utilizing pheromone impregnated lures is available for approximately 11 U.S. dollars per protected tree (SPLAT Verb, ISCA^®^ Technologies, Riverside, CA, USA).

The field application of the aggregation pheromone would likely have no adverse impacts or non-target effects. Low concentrations of the aggregation pheromone are present in the foliage of *Tamarix*, giving it a ubiquitous presence in stands of *Tamarix*^20^. The aggregation pheromone is specific to *Diorhabda* species, with low likelihood of its application interfering with other organism’s pheromone systems. Giving land managers a tool to direct *D. carinulata* around in the field would also allow for land managers to minimize indirect effects from the insects. If a detrimental indirect effect is being observed, land managers can now redirect *D. carinulata* to alternate areas to minimize that detrimental interaction. By drawing beetles away from some areas where *Tamarix* is thought to provide transient ecosystem services, the use of pheromones could have broad implications for conservation, as areas sensitive to the removal or damage of *Tamarix* could be avoided, while intensifying impacts in desired areas^19,25^. The application of this technology could be beneficial to the conservation of native habitat. For example, *Tamarix* commonly dominates landscapes, but there is usually a remnant population of native vegetation present within *Tamarix* infestations^4,26^. Applying the aggregation pheromone to increase *D. carinulata* herbivory on *Tamarix* plants surrounding remnant native plants could remove the competition exerted by *Tamarix* with minimal risk or disturbance to the native plants. Conditions facilitating recruitment of cottonwoods (*Populus* spp.) and willows (*Salix* spp.) are also commonly associated with the successful recruitment of *Tamarix*, resulting in seedling nurseries that are composed of both the native plants and *Tamarix*^27,28^. Selective removal of *Tamarix* seedlings from these mixed species nurseries, whether through herbicide application or mechanical removal, is time-consuming and all but impossible to accomplish, considering that *Tamarix* seedlings can reach densities as high as 170,000 individuals/m^2^ on mud flats^29^. If land managers used pheromone lures to increase *D. carinulata* aggregations on mixed native/*Tamarix* nurseries, the host specificity of the agent would confer a competitive advantage on non-*Tamarix* seedlings. Increased mortality of *Tamarix* during seedling establishment has the potential to reduce long term negative environmental impacts of *Tamarix* invasion, such as salt accumulation and altered fire regimes, on the viability and diversity of affected native plant communities^30,31^.

The ability to manipulate the spatial distribution of *D. carinulata* has biological control applications beyond the enhancement of feeding on *Tamarix*. By applying the aggregation pheromone, and manipulating the densities of *D. carinulata* in targeted areas, dispersal and establishment can be readily assessed. Specifically, the aggregation pheromone can be used to implement a monitoring procedure where plants are treated and act as ‘sentinels’ for the presence of *D. carinulata*^32^. Treated sentinel plants would allow for easy monitoring of *D. carinulata*, especially if combined with passive yellow sticky card or delta traps^12,20^.

## Material and Methods

### Field experiment location and insect source

*Tamarix* stands along the Bighorn River near Lovell, WY were used as the study area during the summer of 2014, 2015, and 2016. Locations were selected based on existing infestations of saltcedar and the presence of *D. carinulata* populations. The *D. carinulata* populations present in the study area are bivoltine and are the descendants of individuals originally collected near the town of Fukang, Xinjiang Province, in northwestern China^10,33^.

### Lure and treatments

Two types of lures were prepared for field testing using ISCA^®^ Technologies’ wax emulsion matrix SPLAT. The first type of lure, hereafter referred to as PH, consisted of SPLAT impregnated with (2*E*,4*Z*)-2,4-heptadien-1-ol, an aggregation pheromone produced by *D. carinulata*^12^. The pheromone was synthesized according to Petroski^34^. The second type of lure, hereafter referred to as BL, consisted of the base formulation of SPLAT with no active ingredient, which functioned as a control treatment. Semiochemical active ingredient (2*E*,4*Z*)-2,4-heptadien-1-ol was present at a concentration of 2.17% in PH treatment lures, and 0% in BL control lures.

The use of 4-g doses of SPLAT, referred to as dollops, facilitated one-time per generation application of SPLAT lure treatments. Treatments were applied to coincide with the emergence of (1) overwintered reproductive adults (P generation); (2) the F1 generation, which were also reproductive; and (3) the F2 generation, which are non-reproductive but actively feed until entering overwintering diapause. In 2014, the first application of SPLAT was June 16 (week 1), the second application of SPLAT was July 16 (week 5), and the final application of SPLAT was August 18 (week 10). In 2015, the first application of SPLAT was May 26 (week 1), the second application of SPLAT was July 13 (week 8), and the final application of SPLAT was August 18 (week 13).

### Release rate analysis

To determine the release rate of the pheromone from 4-g dollops, individual dollops were applied to cattle ear tags and exposed to outdoor conditions at the Bozeman (Montana) Forestry Sciences Laboratory (USDA Forest Service, Rocky Mountain Research Station). Cattle ear tags (Y-Tex Corporation, Cody, WY, USA) were used as the application substrate for dollops because of their durability, portability, and general ease of handling. Volatile collections from these field aged dollops were made at 1, 3, 5, 10, 15, 25, and 31 days to characterize pheromone release rates. Dollops were temporarily transported from the outdoors to the laboratory for volatile collection, but were otherwise continuously exposed to outdoor conditions. Collections were made in glass volatile collection chambers for varying durations depending on the length of field exposure (15 min for samples aged 1, 3, 5, and 10 d; 30 min at 15 d; and 1 hr at 25 and 35 d). Volatile collection traps containing 30 mg of super-Q (Alltech Associates, Inc., Deerfield, IL, USA) adsorbent were fixed in place at the apical opening of the volatile collection chamber where purified air was delivered at a rate of 100 ml/min. Collected volatiles were eluted from the traps into vials using 200 µl methylene chloride (purity >99%) (Fischer Scientific, Pittsburg, PA, USA) and the samples were spiked with 10 µl of a 0.84 ngµl^−1^ solution of 1-octanol (purity >99%) (Sigma-Aldrich, St. Louis, MO, USA) in methylene chloride as an internal standard. Volatiles were analyzed using a gas chromatograph (Agilent 6890; Agilent Technologies, Santa Clara, CA) coupled to a mass selective detector (MSD, Agilent 5973 instrument). Quantification was made relative to the internal standard.

### Field study

The existing arrays of *Tamarix* plants were randomly assigned treatments at two study sites located in a greasewood (*Sarcobatus vermiculatus* Hook) floodplain of the Bighorn River characterized as having average *Tamarix* densities of 0.65 plants/m^2^ ^35^. The study sites were located approximately 1 km apart.

Twelve replicates of each treatment (PH and BL) were deployed on the study sites. Dollops of SPLAT were applied to cattle ear tags using an 80-ml syringe. Application of 5 ml of either the semiochemical or control formulations of SPLAT yielded 4-g dollops. Treated ear tags were attached with a loop of wire to the branches of treatment plants. Plants selected to receive a SPLAT treatment were based on the existing array of plants at the research locations, with a requirement of treated plants to be approximately 20 m apart. This distance has been experimentally confirmed to minimize possible interference between volatile treatments^12,20^. Plant size and age class was allowed to vary with the treatments, with plants canopies as small as 4 m^3^ and canopies as large as 150 m^3^ receiving treatments. The average canopy volume of BL-treated plants was 34.9 m^3^, while the average canopy volume of PH-treated plants was 35.7 m^3^.

Treated *Tamarix* plants were sampled weekly using a heavy duty canvas sweep net that had an opening diameter of 30.5 cm and a depth of 61 cm. Treated plants were swept three times with a 1.25 m upward arc^36^. After sweeping, the captured *D. carinulata* adults and larvae were counted and returned to the tree where they originated. Reproductive status of captured adults was assessed for subsequent analysis. Determination of reproductive status was based on the condition of the ovaries, ovarioles, and accessory glands discerned from dissected adults^13^. Adults used for dissections were collected adjacent to the research locations.

Damage due to *D. carinulata* was visually rated as the percentage of *Tamarix* foliar tissue damaged and was measured in increments of 5%^37^. Feeding by adults or larvae causes affected foliage to turn brown, wither, and frequently die. Plants that were completely brown are considered to be 100% damaged, even if the withered foliage is still attached to the plant. *Tamarix* can recover from defoliation by *D. carinulata* and can even regrow foliage within the same season as the defoliation event. If regrowth of the *Tamarix* plants was observed, the percentage regrowth was visually estimated and the percentage was added as non-damaged green foliage.

Percent dieback and canopy volume were determined once during the field seasons of 2015 and 2016 for each treated plant. Dieback was recorded as the percentage of branches with live foliage the previous year that did not produce foliage in the current year. Evaluation of dieback was made well after *Tamarix* bud burst to allow enough time for new growth to occur. Branch color, flexibility and the presence or absence of foliage were used to categorize individual branches as dead or alive. Canopy volume was recorded in the fall, to account for possible regrowth and recovery of the *Tamarix* plants. Canopy volume was estimated using a formula for a cube. Two perpendicular measurements were taken at the plant’s widest points to estimate canopy length and width, and another measurement was taken at the tallest point to estimate canopy height.

### Statistical analysis

Data from the two research sites were pooled for analysis. A repeated measure ANOVA, using within-year sampling date as the repeated measure, and SPLAT formulation applied (PH vs. BL) as the treatment factor, was used to evaluate for potential differences in adult *D. carinulata* capture densities. Data were ln(x + 1) transformed to better meet the assumptions of normality. Data on *D. carinulata* adults were segregated for analysis according to reproductive or non-reproductive status of the sampled cohort.

Damage rating was similarly assessed using a repeated measures ANOVA, with sampling day as the repeated measure and SPLAT application as the treatment factor. Potential changes in canopy volume were evaluated using a repeated measure ANOVA with year as the repeated measure and SPLAT application as the treatment factor. Canopy volume was ln(x + 1) transformed to better match the assumptions of normality. Repeated measure ANOVAs used type III error and Satterthwaite approximation for degrees of freedom. Percentage dieback was analyzed using a one-way ANOVA. ANOVAs were conducted using the lmer function in the lmerTest package of R software version 3.1.2^38^.

### Disclaimer

Mention of trade names or commercial products in this publication is solely for the purpose of providing specific information and does not imply recommendation or endorsement by the U.S. Department of Agriculture (USDA). USDA is an equal opportunity provider and employer.

## Supplementary information

Supplemental Information

Supplemental Data set

## Data Availability

All data analyzed during this study is included in Supplementary Information files.
