# Supplementary material for: Field demonstration of a semiochemical treatment that enhances Diorhabda carinulata biological control of Tamarix spp
Source: Sci Rep. 2019 Sep 10;9:13051. doi: 10.1038/s41598-019-49459-5 (PMC6737090; doi:10.1038/s41598-019-49459-5)
Supplement: Supplementary file 1 — Supplemental Information [file 41598_2019_49459_MOESM1_ESM.pdf]

Field demonstration of a semiochemical treatment that enhances *Diorhabda carinulata* biological control of *Tamarix* spp.

Alexander M. Gaffke[1,2], Sharlene E. Sing[3], Tom L. Dudley[4], Daniel W. Bean[5], Justin A. Russak[6], Agenor Mafra-Neto[7], Robert K. D. Peterson[1], David K. Weaver[1]*

[1]Department of Land Resources and Environmental Sciences, Montana State University, Bozeman MT, 59717, USA

[2]Agricultural Research Service, United States Department of Agriculture, Center for Medical, Agricultural, and Veterinary Entomology, Gainesville FL, 32608, USA

[3]USDA Forest Service, Rocky Mountain Research Station, Bozeman MT, 59717, USA

[4]Marine Science Institute, University of California, Santa Barbara CA 93106, USA

[5]Colorado Department of Agriculture, Palisade Insectary, Palisade CO, 81526, USA

[6]Department of Chemistry and Biochemistry, University of California Santa Barbara, Santa Barbara CA, 93106, USA

[7]ISCA Technologies, Inc., Riverside CA 92507, USA

***Correspondence to: David K. Weaver, Montana State University, P.O. Box 173120, Bozeman, MT, 59717. Email: weaver@montana.edu

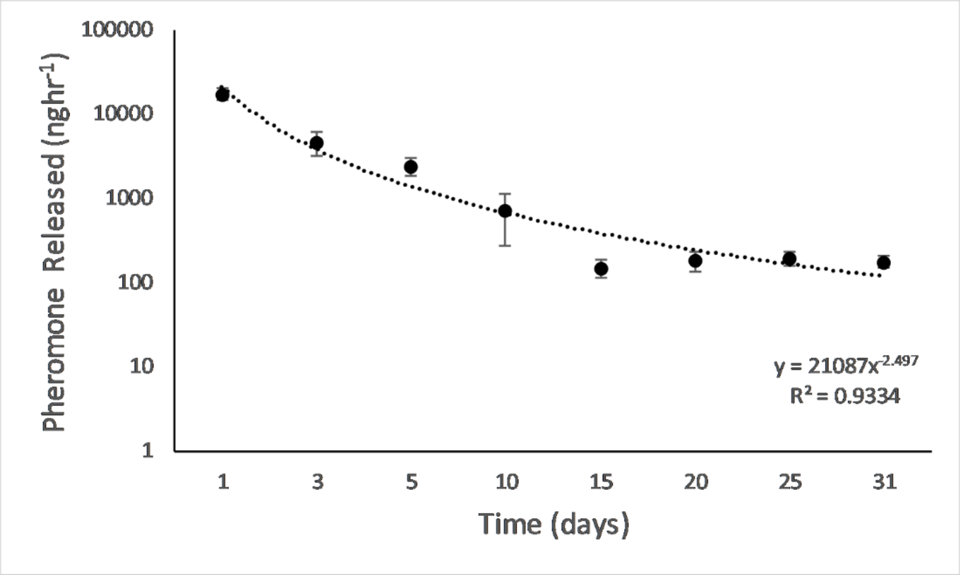

Fig. S1 Mean ± SE of (2*E*, 4*Z*)-2,4-heptadien-1-ol emitted from 4-g dollops.
